# Case Report: Interferon- γ Rescues Monocytic Human Leukocyte Antigen Receptor (mHLA-DR) Function in a COVID-19 Patient With ARDS and Superinfection With Multiple MDR 4MRGN Bacterial Strains

**DOI:** 10.3389/fimmu.2021.753849

**Published:** 2021-11-01

**Authors:** Clemens Grimm, Steffen Dickel, Julian Grundmann, Didier Payen, Julie Schanz, Andreas Erich Zautner, Björn Tampe, Onnen Moerer, Martin Sebastian Winkler

**Affiliations:** ^1^ Department of Anesthesiology and Intensive Care Medicine, University Medical Center Göttingen, Göttingen, Germany; ^2^ Université Paris 7 Cité Sorbonne, UMR INSERM 1160, Paris, France; ^3^ Institute of Clinical Chemistry and Laboratory Medicine, University Medical Center Göttingen, Göttingen, Germany; ^4^ Department of Medical Microbiology and Virology, University Medical Center Göttingen, Göttingen, Germany; ^5^ Department of Nephrology and Rheumatology, University Medical Center Göttingen, Göttingen, Germany

**Keywords:** immune response, mHLA-DR, infection, bacterial, complications, septicemia, MDR, interferon

## Abstract

**Background:**

CD14+ monocytes present antigens to adaptive immune cells *via* monocytic human leukocyte antigen receptor (mHLA-DR), which is described as an immunological synapse. Reduced levels of mHLA-DR can display an acquired immune defect, which is often found in sepsis and predisposes for secondary infections and fatal outcomes. Monocytic HLA-DR expression is reliably induced by interferon- γ (IFNγ) therapy.

**Case Report:**

We report a case of multidrug-resistant superinfected COVID-19 acute respiratory distress syndrome (ARDS) on extracorporeal membrane oxygenation (ECMO) support. The resistance profiles of the detected *Klebsiella pneumoniae*, *Pseudomonas aeruginosa, Acinetobacter baumannii and Citrobacter freundii isolates* were equipped with resistance to all four antibiotic classes including carbapenems (4MRGN) and Cefiderocol in the case of *K. pneumoniae*. A causal therapeutic antibiotic strategy was not available. Therefore, we measured the immune status of the patient aiming to identify a potential acquired immune deficiency. Monocyte HLA-DR expression identified by FACS analysis revealed an expression level of 34% positive monocytes and suggested severe immunosuppression. We indicated IFNγ therapy, which resulted in a rapid increase in mHLA-DR expression (96%), rapid resolution of invasive bloodstream infection, and discharge from the hospital on day 70.

**Discussion:**

Superinfection is a dangerous complication of COVID-19 pneumonia, and sepsis-induced immunosuppression is a risk factor for it. Immunosuppression is expressed by a disturbed antigen presentation of monocytes to cells of the adaptive immune system. The case presented here is remarkable as no validated antibiotic regimen existed against the detected bacterial pathogens causing bloodstream infection and severe pneumonia in a patient suffering from COVID-19 ARDS. Possible restoration of the patient’s own immunity by IFNγ was a plausible option to boost the patient’s immune system, eliminate the identified 4MRGNs, and allow for lung recovery. This led to the conclusion that immune status monitoring is useful in complicated COVID-19-ARDS and that concomitant IFNγ therapy may support antibiotic strategies.

**Conclusion:**

After a compromised immune system has been detected by suppressed mHLA-DR levels, the immune system can be safely reactivated by IFNγ.

## Introduction

Acute lung injury induced by *severe acute respiratory syndrome coronavirus type 2* (SARS-CoV-2) is often complicated by the occurrence of secondary infections (1). Despite adequate antimicrobial treatment, bacterial clearance has been shown to be impaired by induced acquired immunosuppression syndrome (AIS) in acute inflammation, including in coronavirus disease (COVID-19) (2). This AIS is characterized by an alteration in antigen presentation for lymphocytes, which can be readily assessed by human leukocyte antigen receptor (HLA-DR) expression (3). A sharp decrease in monocyte human leukocyte antigen receptor (mHLA-DR) expression was observed in COVID-19 intensive care patients (4, 5), which was associated with changes in interferon gene expression by SARS-CoV-2 (6, 7). Secondary bacterial infections in critically ill COVID-19 patients increase morbidity and mortality (8). We report a COVID-19 patient suffering from pulmonary superinfection with multiple multidrug-resistant (MDR) pathogens who was treated with interferon-γ (IFNγ), resulting in a rapid increase in mHLA-DR expression and a transient improvement in clinical condition.

## Medical History

A 40-year-old patient without specific risk factors or pre-existing comorbidities required ICU treatment for respiratory failure related to SARS-CoV-2 infection. Despite initial treatment with remdesivir and tocilizumab, the patient developed severe ARDS requiring invasive ventilation on day 9 after admission. After continued clinical deterioration, veno-venous extracorporeal membrane oxygenation (ECMO) was implanted on day 11 before the patient was transferred to our ARDS/ECMO center.

## Clinical Course

**Figure 1:**
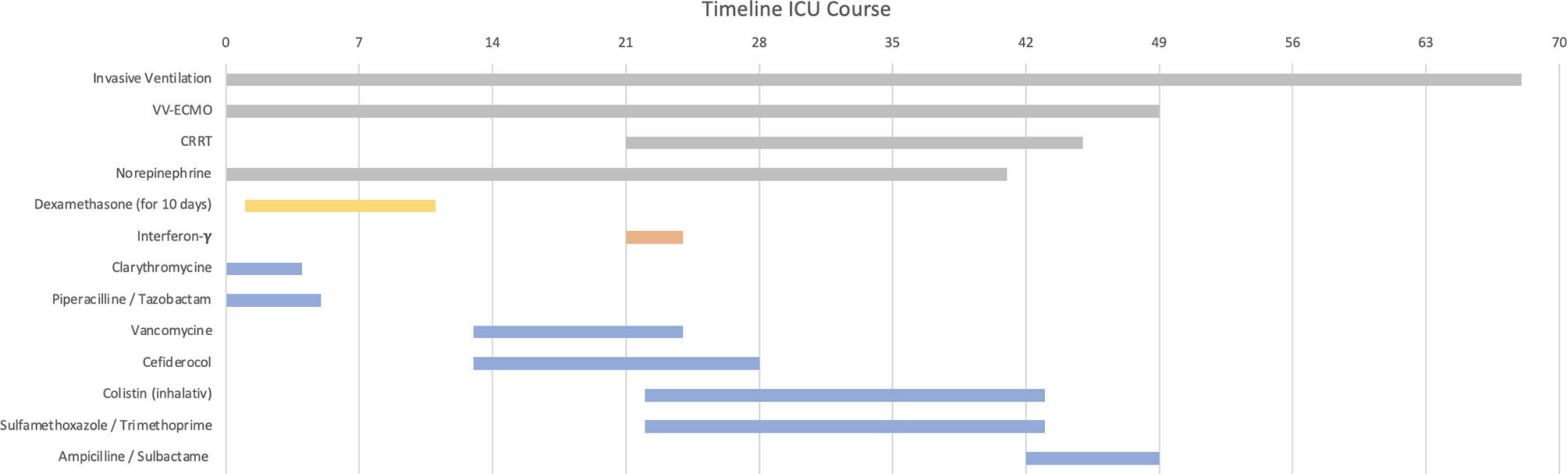
Patients clinical course from admission to our university medical center intensive care unit (ICU). Dexamethasone therapy is shown in yellow; Interferon-y-therapy is shown in red and antibiotics are shown in blue.

After admission to our ICU, different ventilatory modes, prone position, neuromuscular blockade and nitric oxide were applied to improve gas exchange. A high ECMO gas flow and output was required (with maximum of 6.5 L/min blood flow, 9L gas flow and 1.33µg/kg/min noradrenaline on the fifth week after admission). We performed continuous renal replacement therapy (CRRT) due to acute kidney injury (AKI) starting at week 3 and lasting 24 days. The longitudinal clinical course (see **Figure 1**) and main immune system parameters (see **Table 1**) are shown. The diagnosis of severe sepsis was confirmed by the detection of multiple bacterial species: four different multidrug-resistant 4MRGN species were detected in blood cultures (*Klebsiella pneumoniae)* and in tracheal fluids (*Pseudomonas aeruginosa, Acinetobacter baumanii, Enterococcus faecalis)* (see **Figure 1** and **Table 2**).

**Table 1:** Clinical findings and laboratory markers of the patient after ICU admission in our ARDS center.

Week		1	2	3	4*	5	6	7	8	9	10	11
Days		0-7	7-14	14-21	21-28	28-35	35-42	42-49	49-56	56-63	63-70	
**Laboratory findings**												
**HLA (CD3+)**	%				22.8	36.0	41.1	49.5	43.1			
**M-HLA (CD14+)**	%				33.7	96.5	84.3	96.9	82.5			
**Monocytes**	%											8,4
**Leukocytes**	10^3^/µl	18.9	19.2	11.5	11.9	13.6	18.3	23.1	10.6	19.7	11.2	7.2
**CRP**	mg/L	3.0	44.6	296.4		267.0				63.4		7.9
**PCT**	µg/L	0.2	0.1	0.5	7.3	3.1	56.0	5.3	0.8	0.2	0.1	0.1
**Clinical findings**												
**SOFA**		5	7	7	11	12	11	10	10	6	5	5
**Norepinephrine**	µg/kg/min	0.11		0.12	0.67	0.15	0.15	0.12	0.48			
**Horowitz**		166	200	250	123	110	330	330	330	353	400	420

*Time of IFNγ administration.

**Table 2:** Bacterial infections of the patient with antibiogram.

	*P. aeruginosa*	*K. pneumoniae*	*K. pneumoniae*	*K. pneumoniae*	*A. baumannii*	*C. freundii*
		PFGE Type 1	PFGE Type 2	PFGE Type 3		
	4 MRGN	4 MRGN	4 MRGN	4 MRGN	4 MRGN	4 MRGN
	TS NO TH RE BS	TS TH RE CL BS	TS TH	TS TH	NO TS	TS TH RE
**Penicillins**	R	R	R	R	R	R
**Cephalosporins**	R	R	R	R	R	R
**Carbapenems**	R	R	R	R	R	R
**Fluoroquinolones**	R	R	R	R	R	R
**Amikacin**	R	R	R	R	I	R
**Cefiderocol**	–	R	S	R	–	–
**Colistin**	S	S	S	S	S	S
Azithromycin*	R	R	S	R	R	S
**Cotrimoxazole**	R	R	R	R	R	R
**Fosfomycin**	R	R	R	R	R	R
**Gentamicin**	R	R	R	R	R	R
Tetracycline*	R	S	S	S	R	S
**Tigecycline**	R	S	S	S	R	S
**Tobramycin**	R	R	R	R	R	R

PFGE, pulsed-field gel electrophoresis; TS, Tracheal secretion; NO, Nose; TH, Throat; RE, Rectal; BS, Blood Stream; CL, Central Line Catheter. *no breakpoints available, only epidemiological cut off values.

## Diagnostics

**Figure 2:**
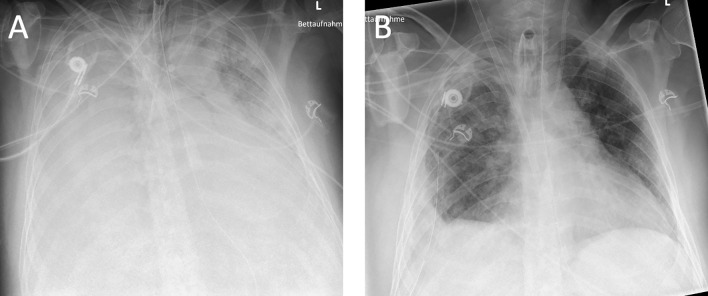
Chest X-ray of the patient immediately before interferon administration at week 4 **(A)** and after administration and clinical improvement at week 6 **(B)**.

Chest radiographs and computed tomography scans revealed severe persistent ARDS with extensive pulmonary infiltrates (See **Figure 2**). The host response was assessed using standard laboratory controls such as *c-reactive protein* (CRP) and *procalcitonin* (PCT). The number of CD14+ monocytes and their HLA-DR expression (33.7%) (measured by flow-cytometry; FACS; measured on a Navios^®^ System, Beckmann Coulter, Brea, CA, USA) (see **Table 1**) was consistent with the diagnosis of a severe immunosuppression syndrome associated with COVID-19.

## Intervention

The observed severe AIS and limited options for antimicrobial therapy made IFNγ a viable option for restoring immune system function. A subcutaneous injection of 100 mcg IFNγ was administered daily for three days at week 4 and was accompanied by clinical and flow cytometric monitoring. Prior written informed consent was obtained from the patient’s legal representative.

## Outcome/Results

No direct adverse effects of IFNγ treatment were observed. CD14+ HLA-DR+ status showed a sharp increase in mHLA-DR after IFNγ administration (see **Table 1**). mHLA-DR increased to a maximum of 96.5% after the initial IFNγ treatment, followed by a slight decrease to 82.9% at the end of the ICU stay.

As shown by the SOFA score, the patient’s clinical condition gradually improved (see **Table 1**), allowing vasopressor infusion to be discontinued on day 41 and ECMO therapy to be stopped on day 49. Ventilator weaning was completed on day 68, and the patient was transferred to the medical ward on day 70 after admission.

## Discussion

This case reports a 40-year-old healthy man who suffered from severe COVID-19 ARDS that required extensive intensive medical care. The resistance profiles of the detected invasive bacterial species *Klebsiella pneumoniae* (three different pulsed-field gel electrophoresis (PFGE) types), *Pseudomonas aeruginosa*, *Acinetobacter baumannii*, and *Citrobacter freundii* left very limited antimicrobial options, warranting an investigation of the potential for immunomodulation by IFNγ. The observed AIS with low expression of HLA-DR in circulating monocytes prompted us to apply IFNγ therapy as in previously described cases (9–11). This resulted in a significant increase in mHLA-DR levels. In addition, there was a slight increase in lymphocytes (CD3+ cells). However, HLA expression in lymphocytes has been shown to be increased as a marker of cell activation in COVID-19 even without prior external administration of IFNγ (12). Nevertheless, it can be assumed that the administration of IFNγ had an additional effect.

In septic patients, we have previously shown that IFNγ treatment altered the number of NK cells in the blood (10). Although not measured in our case, a possible influence of IFNγ on the number and function of NK cells and on the resolution of infection is conceivable (13).

There is also evidence that interferon may have an antiviral effect. For this reason, it cannot be excluded that the viral load was also reduced by IFNγ. Indeed, one *in-vitro* study showed significantly reduced viral replication in SARS-CoV. An additional antiviral effect *in-vivo in SARS-CoV-2* is therefore possible (14).

Immune stimulation by IFNγ in our case was similar to that reported previously in another infectious context and appears to be efficient in severe COVID-19 (9–11). AIS has been described in COVID-19 patients in the ICU, but few data on immunostimulation have been reported to date. Overall, the level of expression of mHLA-DR appears to be a reliable marker for the diagnosis of sepsis-induced immunosuppression (4). Because AIS is not always present in severe COVID-19, the syndrome must be diagnosed by repeated monitoring of the immune system to determine whether immunostimulation should be performed, thus allowing individualized treatment. The usage of immunosuppressant drugs such as dexamethasone or tocilizumab may increase the rates of AIS encountered in COVID-19 patients. Monitoring the immune status of COVID-19 patients is important and should be a standard of care, as alternative treatment options such as IFNγ therapy are available.

Several case series have shown that administration of IFNγ results in a significant increase in mHLA-DR expression, improves the ability to eliminate invasive bacterial pathogens, and in most cases results in rapid clinical improvement (9–11). These previous reports were convincing enough to motivate randomized clinical trials, which have not yet been reported. The present case is remarkable because it is a desperate case with six highly resistant bacterial isolates from four different microbial species in very severe COVID-19. Immune system support was particularly important in this patient for the regression of his severe infection and for the elimination of the bacteria. Antibiotic therapy options were limited. For example, the bacterial strains were resistant to sulfamethoxazole/trimethoprime and only colistin was available as a last choice. However, it should be noted that colistin is largely filtered by continuous renal replacement therapy (CRRT) and only an inhaled formulation of this drug was available (15). It is possible that colistin contributed to the patient’s recovery, however, it does not explain the increased mHLA-DR levels and the rapid clinical improvement.

MDR bacterial strains, particularly Pseudomonas aeruginosa and Klebsiella pneumoniae, have been shown to be associated with unrestricted antibiotic treatment (16), with 18% of German ICUs using prophylactic antibiotics for COVID-19 patients (17). The misuse of antibiotics is one of the main causes of the increase in infections with multidrug-resistant pathogens (18). In the future, an increasing number of patients suffering from infections with MDR pathogens can be expected. The possibilities of immunomodulation seem to be a viable treatment alternative for severe cases and warrant further investigation (18).

The choice of IFNγ was based on previous experience and on the absence of reported severe side effects. In addition, this drug has limited immune targets on innate immune cells, which may partially result from a deficit in IFNγ secretion by lymphocytes (10). The pharmacokinetics appear to be particularly appropriate for patients in the intensive care unit, as the effect is measurable within the first or second day after initiation of treatment and disappears rapidly after discontinuation (10). The authors hope that further research into how IFNγ supports the immune system will lead to a better understanding of the mechanisms involved, allowing for improved individualized therapies for critically ill patients.

## Limitations

This is a case report of one patient and little can be said about the general response and overall safety of IFNγ therapy. Nevertheless, there are case reports concerning patients suffering from COVID-19 confirming our successful treatment. To improve the quality of this report, we have edited and presented the report according to the CARE criteria (see Equator Network) (19).

## Conclusion

A compromised immune system can be detected by measuring mHLA-DR expression. With the administration of IFNγ, mHLA-DR expression on monocytes was restored without severe side effects. This is particularly important regarding the fact that antibiotic therapy is not possible under certain circumstances. Supporting the body’s own defenses thus represents an additional or, if necessary, alternative option for eliminating infectious agents. However, we would like to point out that further studies on this therapy are needed.

## Data Availability Statement

The raw data supporting the conclusions of this article will be made available by the authors, without undue reservation.

## Ethics Statement

Ethical review and approval were not required for the study on human participants in accordance with the local legislation and institutional requirements. The patients/participants provided their written informed consent to participate in this study. Written informed consent was obtained to describe this case anonymously. This article does not contain any information that allows inference of the patient’s identity.

## Author Contributions

SD, CG, OM, BT, and MW treated the patient. JS performed the laboratory and FACS analysis. AZ performed microbiological diagnostics. CG, SD, DP, BT, and JG analyzed the patient data. CG, SD, and JG wrote the manuscript. DP, JS, AZ, OM, BT, and MW helped to finish the final version of the manuscript. All authors read and accepted the final version of the manuscript.

All authors support the current recommendations of the International Committee of Medical Journal Editors (ICMJE). All authors contributed to the article and approved the submitted version.

## Funding

The publication of the case report was supported by the open access publication fund of the Goettingen University Medical Center.

## Conflict of Interest

The authors declare that the research was conducted in the absence of any commercial or financial relationships that could be construed as a potential conflict of interest.

## Publisher’s Note

All claims expressed in this article are solely those of the authors and do not necessarily represent those of their affiliated organizations, or those of the publisher, the editors and the reviewers. Any product that may be evaluated in this article, or claim that may be made by its manufacturer, is not guaranteed or endorsed by the publisher.

## References

[1] Ripa M Galli L Poli A Oltolini C Spagnuolo V Mastrangelo A. Secondary Infections in Patients Hospitalized With COVID-19: Incidence and Predictive Factors. Clin Microbiol Infect (2021) 27(3):451–7. doi: 10.1016/j.cmi.2020.10.021 PMC7584496 33223114

[2] Shi C Pamer EG. Monocyte Recruitment During Infection and Inflammation. Nat Rev Immunol (2011) 11(11):762–74. doi: 10.1038/nri3070 PMC3947780 21984070

[3] Lukaszewicz A-C Grienay M Resche-Rigon M Pirracchio R Faivre V Boval B. Monocytic HLA-DR Expression in Intensive Care Patients: Interest for Prognosis and Secondary Infection Prediction. Crit Care Med (2009) 37(10):2746–52. doi: 10.1097/00003246-200910000-00011 19707128

[4] Benlyamani I Venet F Coudereau R Gossez M Monneret G. Monocyte HLA-DR Measurement by Flow Cytometry in COVID-19 Patients: An Interim Review. Cytometry Part A (2020) 97(12):1217–21. doi: 10.1002/cyto.a.24249 33125816

[5] Venet F Demaret J Gossez M Monneret G. Myeloid Cells in Sepsis-Acquired Immunodeficiency. Ann N Y Acad Sci (2021) 1499(1):3–17. doi: 10.1111/nyas.14333 32202669

[6] Lei X Dong X Ma R Wang W Xiao X Tian Z. Activation and Evasion of Type I Interferon Responses by SARS-CoV-2. Nat Commun (2020) 11(1):3810. doi: 10.1038/s41467-020-17665-9 32733001 PMC7392898

[7] Tay MZ Poh CM Rénia L MacAry PA Ng LFP. The Trinity of COVID-19: Immunity, Inflammation and Intervention. Nat Rev Immunol (2020) 20(6):363–74. doi: 10.1038/s41577-020-0311-8 PMC7187672 32346093

[8] Langford BJ So M Raybardhan S Leung V Westwood D MacFadden DR. Bacterial Co-Infection and Secondary Infection in Patients With COVID-19: A Living Rapid Review and Meta-Analysis. Clin Microbiol Infect (2020) 26(12):1622–9. doi: 10.1016/j.cmi.2020.07.016 PMC7832079 32711058

[9] Dickel S Grimm C Amschler K Schnitzler SU Schanz J Moerer O. Case Report: Interferon-γ Restores Monocytic Human Leukocyte Antigen Receptor (mHLA-DR) in Severe COVID-19 With Acquired Immunosuppression Syndrome. Front Immunol (2021) 12:645124(1087). doi: 10.3389/fimmu.2021.645124 33897692 PMC8058468

[10] Payen D Faivre V Miatello J Leentjens J Brumpt C Tissières P. Multicentric Experience With Interferon Gamma Therapy in Sepsis Induced Immunosuppression. A Case Series. BMC Infect Dis (2019) 19(1):931. doi: 10.1186/s12879-019-4526-x 31690258 PMC6833157

[11] Nguyen LS Ait Hamou Z Gastli N Chapuis N Pène F. Potential Role for Interferon Gamma in the Treatment of Recurrent Ventilator-Acquired Pneumonia in Patients With COVID-19: A Hypothesis. Intensive Care Med (2021) 47(5):619–21. doi: 10.1007/s00134-021-06377-3 PMC7943696 33688993

[12] Files JK Boppana S Perez MD Sarkar S Lowman KE Qin K. Sustained Cellular Immune Dysregulation in Individuals Recovering From SARS-CoV-2 Infection. J Clin Invest (2021) 131(1). doi: 10.1172/JCI140491 PMC7773371 33119547

[13] Tosello-Trampont A Surette FA Ewald SE Hahn YS. Immunoregulatory Role of NK Cells in Tissue Inflammation and Regeneration. Front Immunol (2017) 8:301(301). doi: 10.3389/fimmu.2017.00301 28373874 PMC5357635

[14] Sainz B Mossel EC Peters CJ Garry RF. Interferon-Beta and Interferon-Gamma Synergistically Inhibit the Replication of Severe Acute Respiratory Syndrome-Associated Coronavirus (SARS-CoV). Virology (2004) 329(1):11–7. doi: 10.1016/j.virol.2004.08.011 PMC7111895 15476870

[15] Markou N Fousteri M Markantonis SL Zidianakis B Hroni D Boutzouka E. Colistin Pharmacokinetics in Intensive Care Unit Patients on Continuous Venovenous Haemodiafiltration: An Observational Study. J Antimicrob Chemother (2012) 67(10):2459–62. doi: 10.1093/jac/dks257 22790220

[16] Sarkar S Khanna P Singh AK. Impact of COVID-19 in Patients With Concurrent Co-Infections: A Systematic Review and Meta-Analyses. J Med Virol (2021) 93(4):2385–95. doi: 10.1002/jmv.26740 33331656

[17] Dickel S Grimm C Popp M Struwe C Sachkova A Golinski M. Infection Control, Prophylactic Antibiotics, and Testing for SARS-CoV-2 and PPE on German Intensive Care Units: Results From a National Mixed Methods Survey. GMS Hyg Infect Control (2021) 16:Doc21. doi: 10.3205/dgkh000392 34194922 PMC8204667

[18] Dunachie SJ Day NPJ Dolecek C. The Challenges of Estimating the Human Global Burden of Disease of Antimicrobial Resistant Bacteria. Curr Opin Microbiol (2020) 57:95–101. doi: 10.1016/j.mib.2020.09.013 33147565 PMC7763986

[19] AGREE. Development and Validation of an International Appraisal Instrument for Assessing the Quality of Clinical Practice Guidelines: The AGREE Project. Qual Saf Health Care (2003) 12(1):18–23. doi: 10.1136/qhc.12.1.18 12571340 PMC1743672

